# Supplementary material for: The Role of Erythrocyte Membrane Protein Band 4.1-like 3 in Idiopathic Pulmonary Fibrosis
Source: Int J Mol Sci. 2023 Jun 15;24(12):10182. doi: 10.3390/ijms241210182 (PMC10298908; doi:10.3390/ijms241210182)
Supplement: Supplementary file 1 [file ijms-24-10182-s001.zip › ijms-2413057-supplementary.pdf]

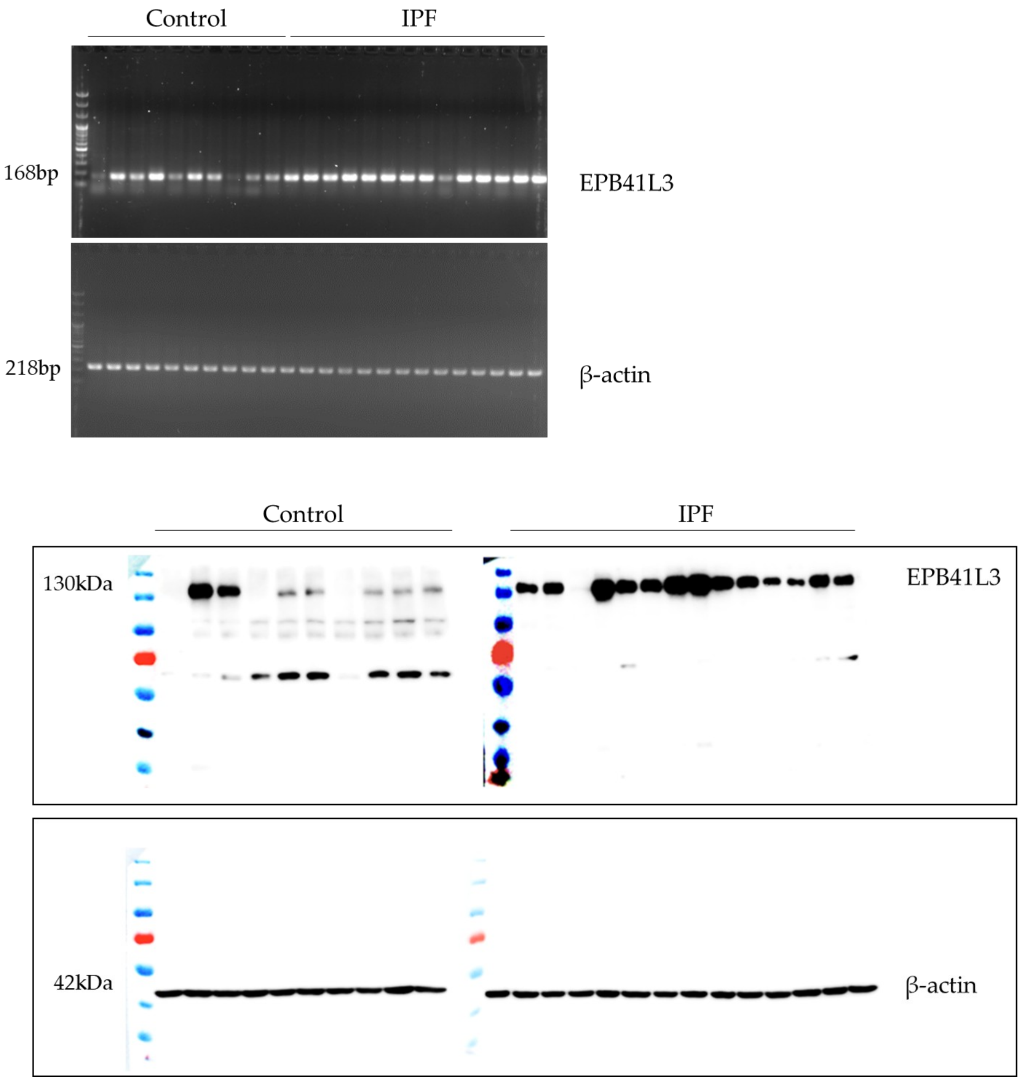

Figure S1. Original RT-PCR and western blot for Figure 1 showing the bands with molecular weight markers.

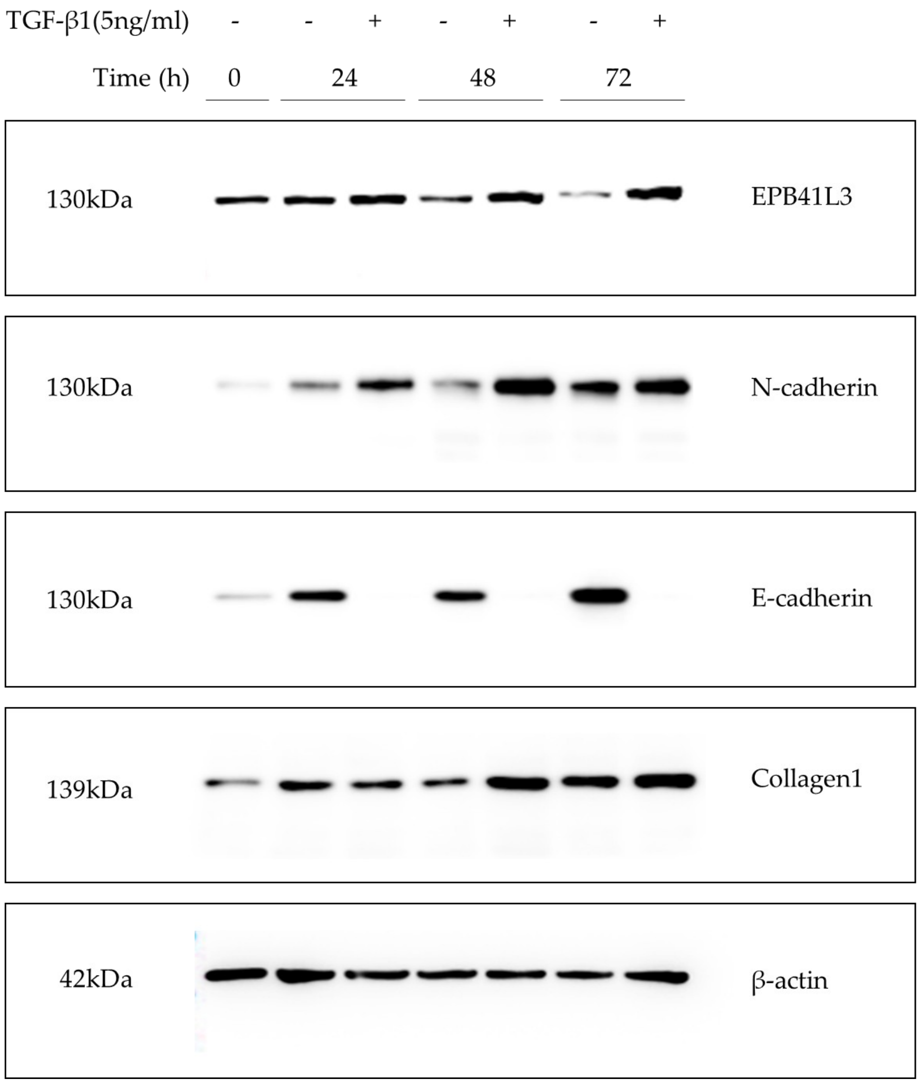

Figure S2. Original Western blot for Figure 2 showing the bands with molecular weight markers.

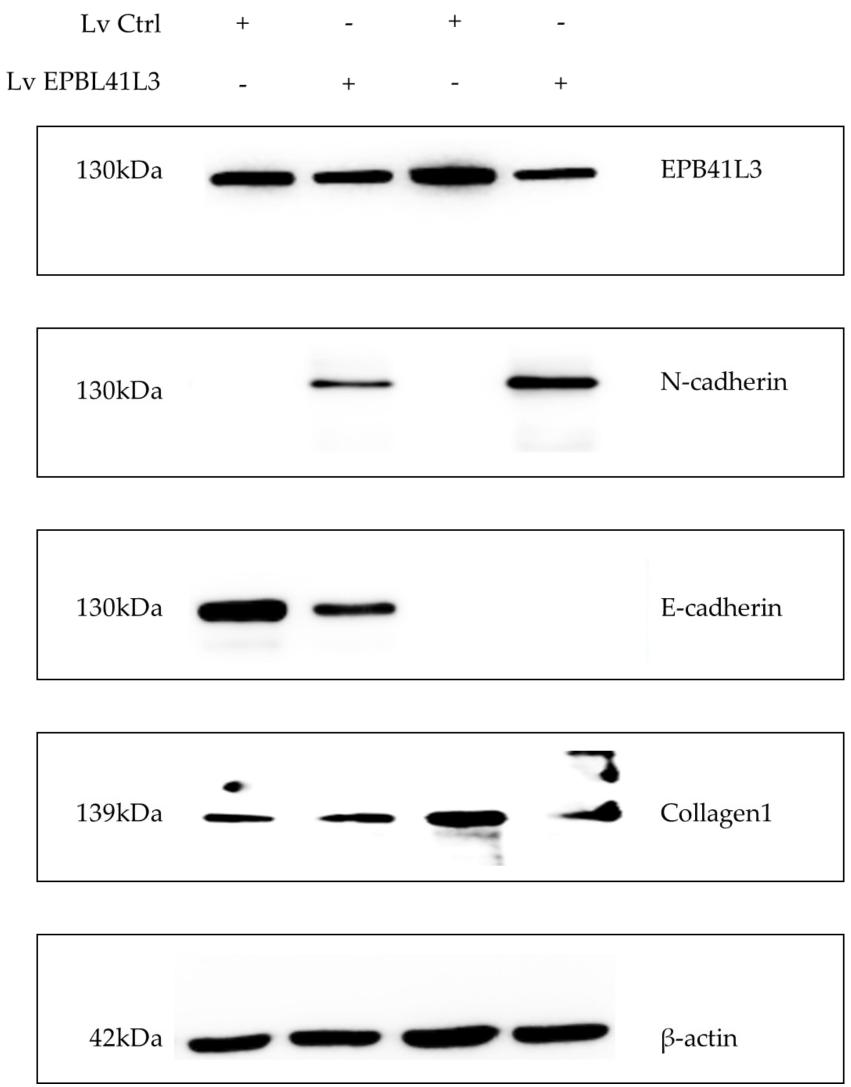

Figure S3. Original Western blot for Figure 3 showing the bands with molecular weight markers.

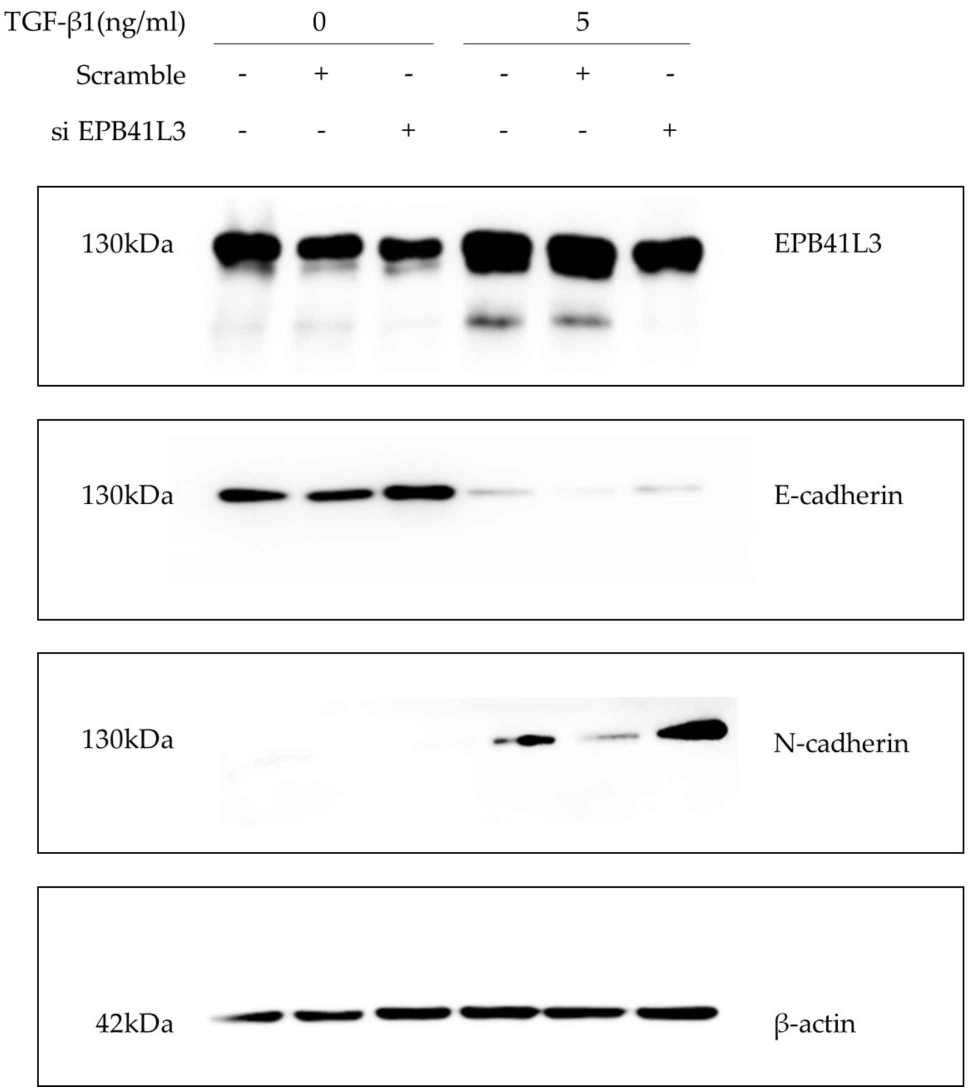

Figure S4. Original Western blot for Figure 4 showing the bands with molecular weight markers.

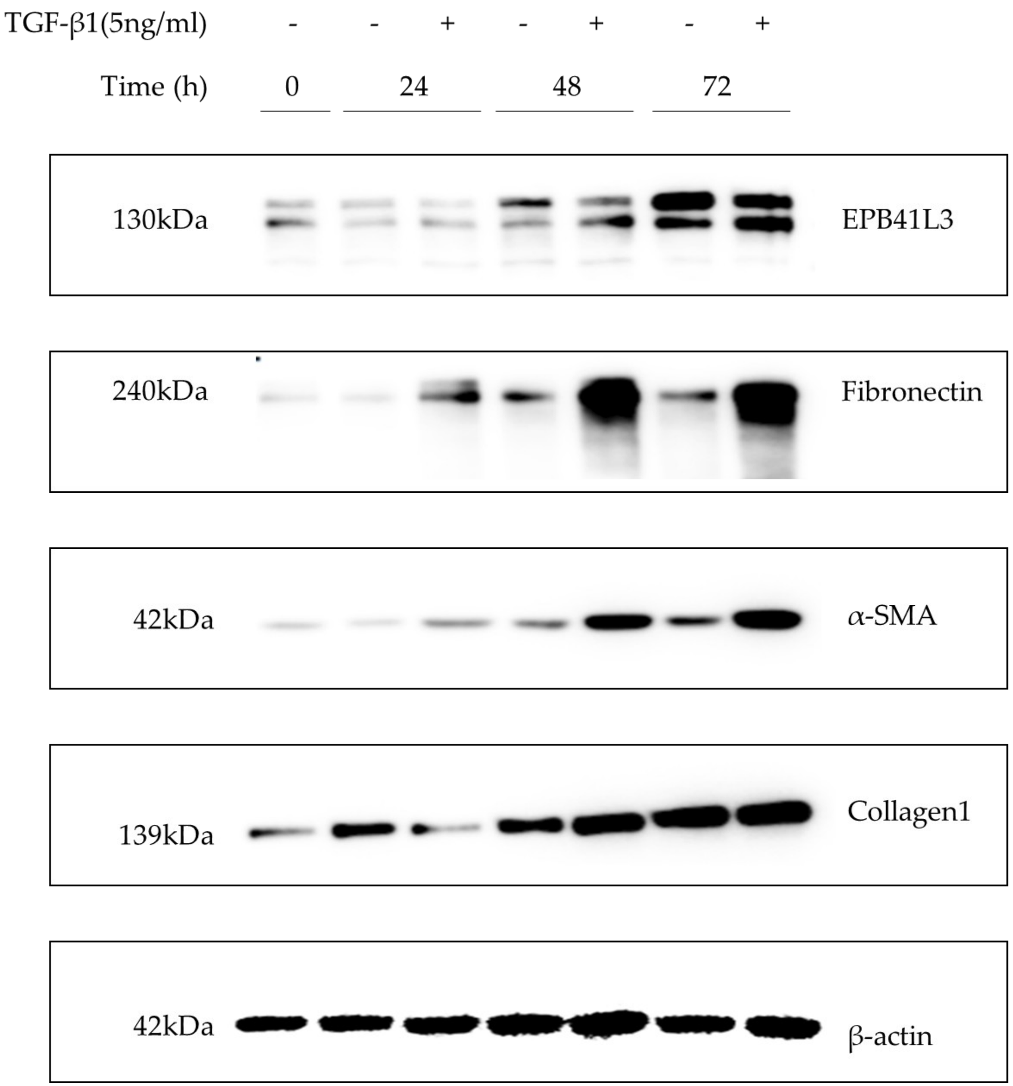

Figure S5. Original Western blot for Figure 5 showing the bands with molecular weight markers.

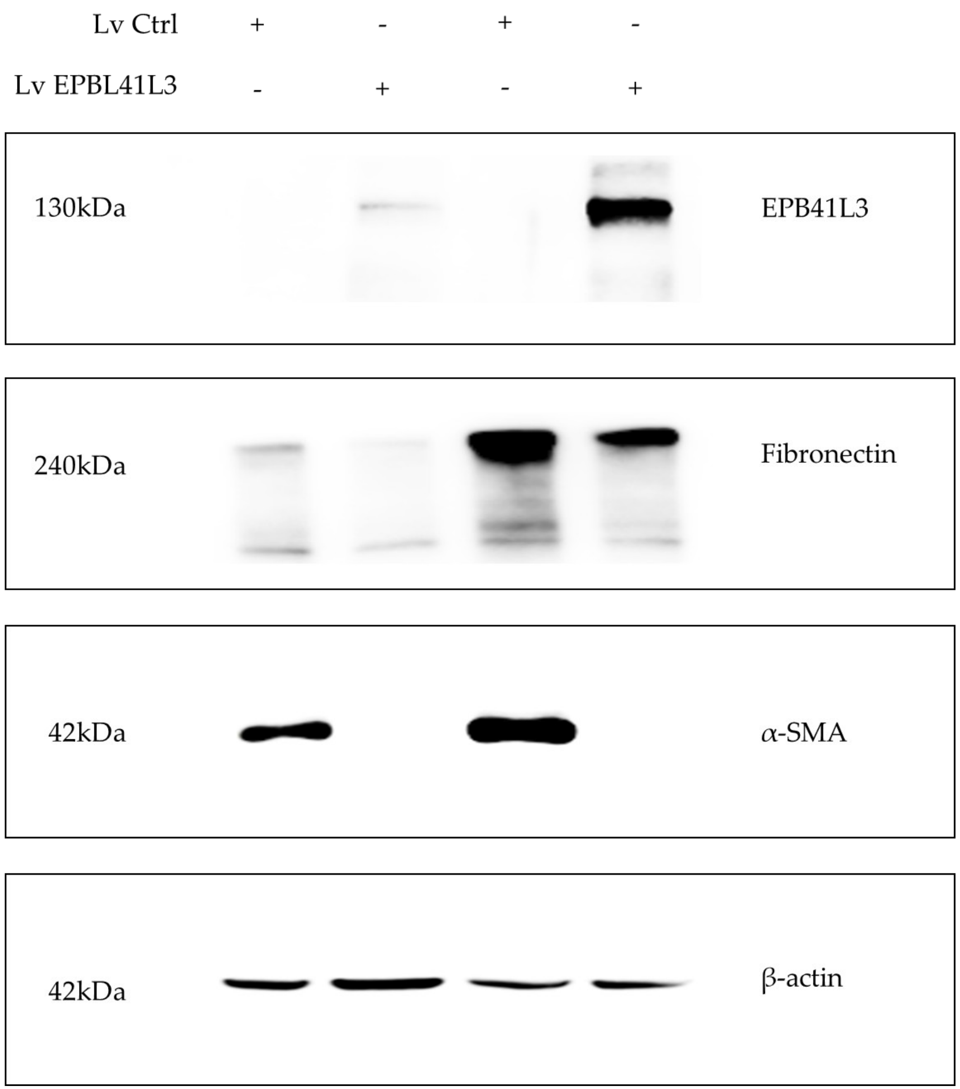

Figure S6. Original Western blot for Figure 6 showing the bands with molecular weight markers.

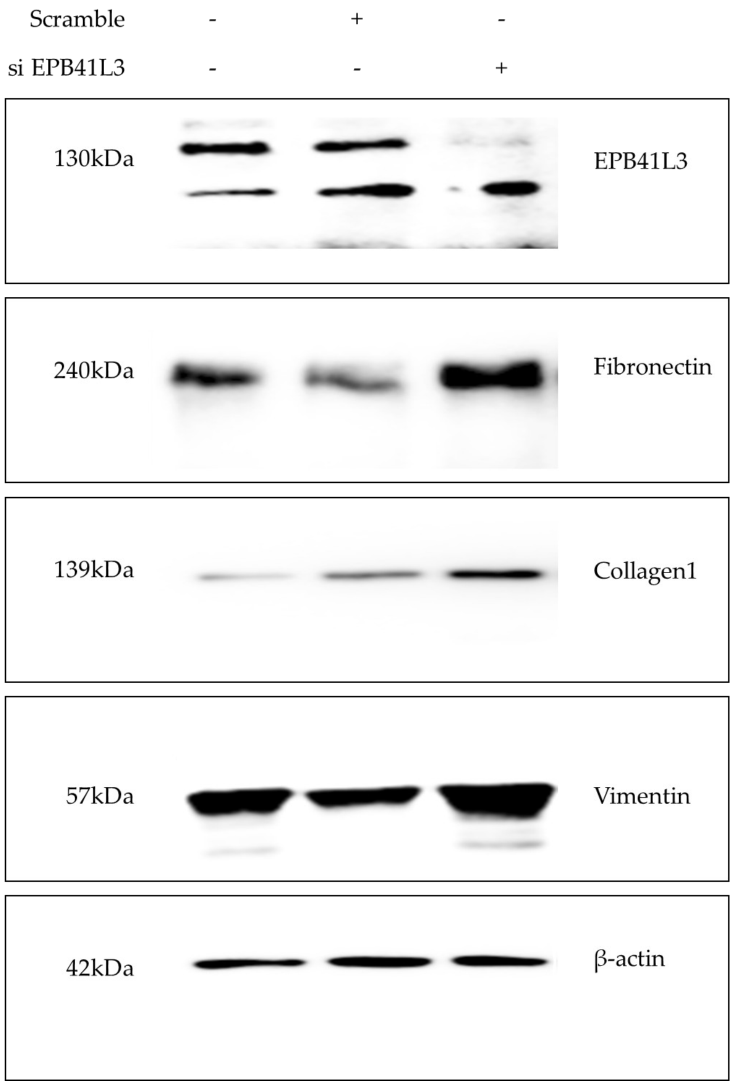

Figure S7. Original Western blot for Figure 7 showing the bands with molecular weight markers.

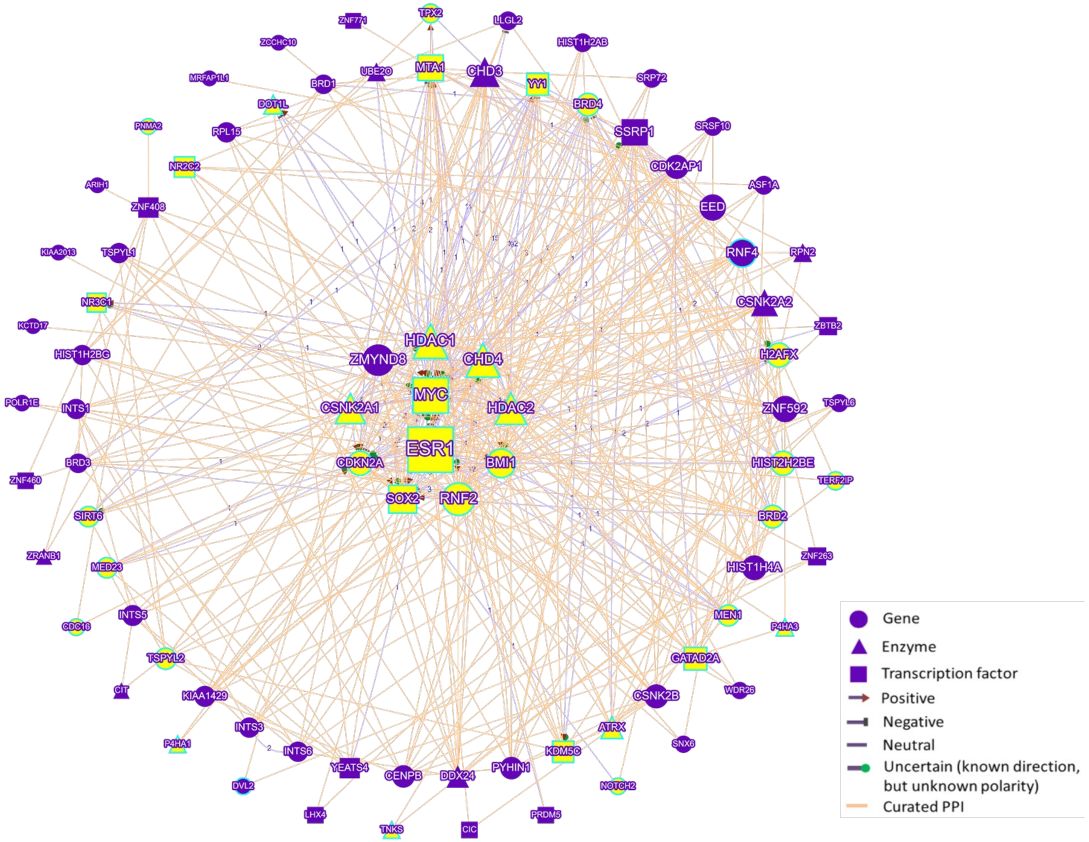

Figure S8. STRING protein-protein interaction network analysis of EPB41L3 and ZNF687 using GenCLiP 3. In the BioGRID database (https://thebiogrid.org/), 99 proteins were identified to interact with ZNF687 in a comprehensive gene regulatory network through text-mining. Circles represent genes, triangles represent enzymes, and transcription factors are shown as squares. The numbers on the interaction lines mean the count of the text mining which has been reported as association. Proteins in yellow-colored boxes are associated with fibrosis.

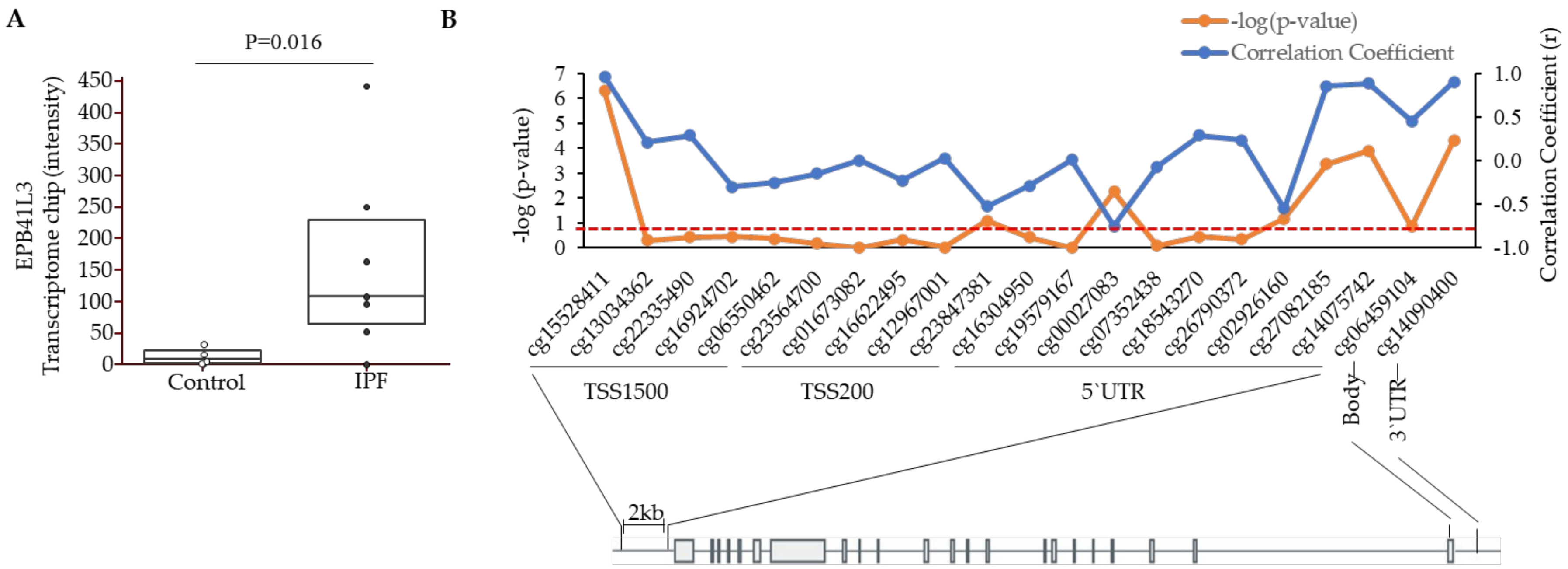

Figure S9. Correlation analysis of EPB41L3 gene expression intensities with CpG DNA methylation levels of 12 fibroblasts.

(A) Comparison of EPB41L3 gene expression levels (GSE71351) between IPF (n = 8) and control fibroblasts (n = 4). (B) CpG methylation levels of 21 loci on EPB41L3 were obtained from GSE107226. Correlation of the CpG methylation levels of 21 CpG loci on EPB41L3 with the gene expression level of the transcriptome chip was presented as correlation coefficient r (right Y-axis) and p-values as - log (left x-axis). The horizontal red line indicates a p-value of 0.05. TSS; transcription start site. UTR; upstream region.

Table S1. List of 344 genes showing positive or negative correlation with *EPB41L3*

Gene	Positive Correlation		Gene	Negative Correlation	
	r	P-value		r	P-value
RASIP1	0.714	0.047	HS.513971	-0.952	0.000
LRRN3	0.714	0.047	HS.255813	-0.929	0.001
HS.336370	0.714	0.047	RPL14	-0.929	0.001
MID2	0.714	0.047	TRAF1	-0.929	0.001
CDH2	0.714	0.047	KLHL13	-0.929	0.001
ZNF286A	0.714	0.047	DKFZP451M2119	-0.905	0.002
DEPDC7	0.714	0.047	ZNRF3	-0.905	0.002
HRES1	0.714	0.047	FOXC1	-0.905	0.002
ADAMTSL5	0.714	0.047	EPC1	-0.905	0.002
RPS27L	0.714	0.047	FOSB	-0.905	0.002
PEAR1	0.714	0.047	LOC642782	-0.903	0.002
C11ORF57	0.714	0.047	ELL	-0.881	0.004
HS.566279	0.714	0.047	CXXC5	-0.881	0.004
ERICH1	0.714	0.047	PARP4	-0.877	0.004
NDUFA4	0.714	0.047	PAPOLG	-0.858	0.006
HPR	0.714	0.047	NDST2	-0.857	0.007
PTGR2	0.714	0.047	UBE2W	-0.857	0.007
PAX7	0.714	0.047	PRR4	-0.857	0.007
LOC391825	0.714	0.047	RGS21	-0.857	0.007
AES	0.714	0.047	HS.442427	-0.857	0.007
PRKCI	0.714	0.047	PHKA1	-0.857	0.007
HS.541852	0.714	0.047	PLCL2	-0.857	0.007
GBP1	0.714	0.047	HS.571515	-0.833	0.010
LOC100132428	0.714	0.047	SCYL1BP1	-0.833	0.010

CYLN2	0.714	0.047	KCTD7	-0.833	0.010
PDLIM7	0.714	0.047	KIAA1383	-0.833	0.010
PKN1	0.714	0.047	ZBTB3	-0.833	0.010
FGR	0.714	0.047	TYW1	-0.833	0.010
COX11P	0.714	0.047	RAMP1	-0.833	0.010
GNB4	0.714	0.047	MED26	-0.833	0.010
PRSS8	0.714	0.047	FLJ32955	-0.810	0.015
LOC729351	0.714	0.047	SNAP29	-0.810	0.015
ZNF687	0.714	0.047	KCNE1L	-0.810	0.015
HS.130455	0.725	0.042	CNIH2	-0.810	0.015
HS.566930	0.725	0.042	HS.545887	-0.810	0.015
FAM46C	0.738	0.037	C11ORF17	-0.810	0.015
CCDC113	0.738	0.037	HS.552087	-0.810	0.015
NCRNA00152	0.738	0.037	ASH1L	-0.810	0.015
EFR3B	0.738	0.037	HS.131259	-0.810	0.015
TSHZ2	0.738	0.037	C2ORF34	-0.810	0.015
HS.541191	0.738	0.037	SOCS2	-0.810	0.015
GNG10	0.738	0.037	PPOX	-0.810	0.015
PFTK1	0.738	0.037	MIR106A	-0.810	0.015
LOC100133222	0.738	0.037	CPOX	-0.810	0.015
LOC645609	0.738	0.037	ARAP1	-0.810	0.015
PISD	0.738	0.037	ZCWPW2	-0.810	0.015
PIK3C2A	0.738	0.037	UPF3B	-0.810	0.015
TTC32	0.738	0.037	PRSS42	-0.810	0.015
CCDC134	0.738	0.037	ZNF439	-0.810	0.015
PPM1A	0.738	0.037	RASD1	-0.810	0.015
LOC344328	0.738	0.037	USP44	-0.810	0.015

TNRC6A	0.738	0.037	LOC729533	-0.810	0.015
ADSS	0.738	0.037	DNAJC5G	-0.808	0.015
IDE	0.738	0.037	LOC647176	-0.792	0.019
PPP2R4	0.738	0.037	LOC100132439	-0.792	0.019
LOC648000	0.738	0.037	HS.547861	-0.788	0.020
MTMR9	0.738	0.037	LOC646938	-0.786	0.021
HS.541654	0.738	0.037	TMEM164	-0.786	0.021
ZNF140	0.738	0.037	CRADD	-0.786	0.021
HCG18	0.738	0.037	LOC100129139	-0.786	0.021
B3GALT2	0.738	0.037	PIAS2	-0.786	0.021
CST7	0.738	0.037	HS.18849	-0.786	0.021
GABRA5	0.747	0.033	PBEF1	-0.786	0.021
COX6B1	0.757	0.030	GMEB1	-0.786	0.021
SPIN2A	0.759	0.029	PTPN3	-0.786	0.021
ARF6	0.762	0.028	CCDC24	-0.786	0.021
HS.547175	0.762	0.028	LOC25845	-0.786	0.021
RNF182	0.762	0.028	ZNF514	-0.786	0.021
KRT7	0.762	0.028	YY1AP1	-0.786	0.021
GPRASP1	0.762	0.028	KIAA1285	-0.786	0.021
MRPL32	0.762	0.028	ZNF143	-0.786	0.021
BCL3	0.762	0.028	PRKRA	-0.786	0.021
KIAA1026	0.762	0.028	NBAS	-0.786	0.021
AP3S1	0.762	0.028	TOP3B	-0.786	0.021
UPK3B	0.762	0.028	ATOH7	-0.786	0.021
C20ORF7	0.762	0.028	TTC8	-0.786	0.021
HS.201950	0.762	0.028	FDPS	-0.786	0.021
ANKS3	0.762	0.028	LOC441662	-0.786	0.021

HS.535718	0.762	0.028	NAP1L6	-0.786	0.021
PTER	0.763	0.028	HS.435043	-0.767	0.026
HS.137971	0.769	0.026	LOC100132106	-0.765	0.027
C17ORF60	0.786	0.021	S100A6	-0.765	0.027
LITAF	0.786	0.021	TREML1	-0.762	0.028
TUBE1	0.786	0.021	HS.565857	-0.762	0.028
HS.44811	0.786	0.021	LOC643505	-0.762	0.028
LOC646853	0.786	0.021	HS.548045	-0.762	0.028
ZNF70	0.786	0.021	FLJ37396	-0.762	0.028
FAT4	0.786	0.021	TMEM127	-0.762	0.028
ELOVL5	0.786	0.021	DDX55	-0.762	0.028
HS.438905	0.786	0.021	ATP6V0A1	-0.762	0.028
LOC283267	0.786	0.021	GAK	-0.762	0.028
FECH	0.786	0.021	ASCL2	-0.762	0.028
PRM3	0.786	0.021	RAB36	-0.762	0.028
LOC100129434	0.786	0.021	PDE6D	-0.762	0.028
RBM34	0.786	0.021	LOC653352	-0.762	0.028
PPP2R2D	0.786	0.021	HS.528210	-0.762	0.028
MT1F	0.786	0.021	FCF1	-0.762	0.028
SLC27A5	0.786	0.021	METTL6	-0.762	0.028
ZER1	0.786	0.021	HS.127398	-0.762	0.028
LOC644640	0.786	0.021	KIAA1731	-0.762	0.028
ZNF131	0.810	0.015	HS.574713	-0.762	0.028
THBS1	0.810	0.015	HS.580154	-0.762	0.028
LOC100130827	0.810	0.015	LOC728216	-0.762	0.028
LOC285053	0.810	0.015	LOC642736	-0.743	0.035
BRI3	0.810	0.015	RELN	-0.738	0.037

TCP10L	0.810	0.015	LOC645416	-0.738	0.037
CALCOCO2	0.810	0.015	HS.580169	-0.738	0.037
MAX	0.810	0.015	C15ORF21	-0.738	0.037
LOC100128291	0.810	0.015	DNAJC25	-0.738	0.037
LOC100129424	0.810	0.015	HS.565863	-0.738	0.037
ADAP2	0.810	0.015	ARSB	-0.738	0.037
TGDS	0.810	0.015	C10ORF84	-0.738	0.037
LOC283767	0.810	0.015	HS.445239	-0.738	0.037
USP32	0.810	0.015	HS.434413	-0.738	0.037
NUDT8	0.810	0.015	ZNF557	-0.738	0.037
OR4D10	0.833	0.010	LOC728211	-0.738	0.037
HS.441813	0.833	0.010	C6ORF138	-0.738	0.037
TEAD3	0.833	0.010	ALDH9A1	-0.738	0.037
OGFRL1	0.833	0.010	C9ORF156	-0.738	0.037
FAHD2B	0.833	0.010	TMEM231	-0.738	0.037
KIAA0802	0.833	0.010	IFT57	-0.738	0.037
TERF2	0.833	0.010	ZNF418	-0.738	0.037
THSD4	0.833	0.010	RPRD1A	-0.738	0.037
MICB	0.833	0.010	ANGEL1	-0.738	0.037
CSRP1	0.833	0.010	DLGAP1-AS2	-0.738	0.037
PPM2C	0.833	0.010	MLST8	-0.738	0.037
LOC100131381	0.833	0.010	ZNF266	-0.738	0.037
C1ORF201	0.833	0.010	VENTXP1	-0.738	0.037
LOC729668	0.833	0.010	ZNF148	-0.738	0.037
LOC100133348	0.857	0.007	COG7	-0.738	0.037
ITGA9	0.857	0.007	TTC5	-0.738	0.037
TCF7L1	0.857	0.007	ARHGEF10L	-0.738	0.037

HS.573359	0.857	0.007	GABARAP	-0.738	0.037
HS.541829	0.857	0.007	TRIOBP	-0.738	0.037
FLJ39827	0.857	0.007	HS.541315	-0.738	0.037
HS.280924	0.857	0.007	C1ORF59	-0.738	0.037
LOC728686	0.857	0.007	ZNF841	-0.738	0.037
LYN	0.881	0.004	QTRT1	-0.738	0.037
FGF2	0.881	0.004	FAM131C	-0.738	0.037
PPIF	0.881	0.004	INADL	-0.738	0.037
SMCP	0.881	0.004	NCF2	-0.738	0.037
C1ORF222	0.881	0.004	LOC648149	-0.725	0.042
THRAP5	0.881	0.004	RSAD2	-0.714	0.047
GPAM	0.881	0.004	GAD1	-0.714	0.047
POLE4	0.881	0.004	PLA2G4C	-0.714	0.047
HS.538089	0.881	0.004	ENPP7	-0.714	0.047
ATP11B	0.881	0.004	HS.527174	-0.714	0.047
AGR3	0.881	0.004	HS.442504	-0.714	0.047
HS.539742	0.881	0.004	LOC729950	-0.714	0.047
HIP1	0.881	0.004	HS.325396	-0.714	0.047
LOC653616	0.905	0.002	HS.545587	-0.714	0.047
WDR5B	0.905	0.002	VPS4B	-0.714	0.047
HS.583022	0.905	0.002	LOC100130837	-0.714	0.047
LOC100134550	0.905	0.002	PPP1R3D	-0.714	0.047
C2ORF63	0.905	0.002	OR2W5	-0.714	0.047
HS.562205	0.905	0.002	ATL2	-0.714	0.047
BRD4	0.905	0.002	FAM134C	-0.714	0.047
EPHB2	0.929	0.001	PIGQ	-0.714	0.047
NMI	0.976	0.000	FASTK	-0.714	0.047

PDHX	0.976	0.000	HS.436627	-0.714	0.047
			PHF21B	-0.714	0.047
			LOC100130552	-0.714	0.047
			MYL1	-0.714	0.047
			FAHD1	-0.714	0.047
			LOC641705	-0.714	0.047
			ZNF768	-0.714	0.047
			TROVE2	-0.714	0.047
			PFN4	-0.714	0.047
			C7ORF49	-0.714	0.047
			HS.46693	-0.714	0.047
			C5ORF35	-0.714	0.047
			LOC93622	-0.714	0.047
			LOC150568	-0.714	0.047
			ARMC7	-0.714	0.047
			RGS12	-0.714	0.047
			HS.551898	-0.714	0.047
			CCNL1	-0.714	0.047
			ANKMY1	-0.714	0.047
			SNX26	-0.714	0.047
			LOC728014	-0.714	0.047
			STX1A	-0.714	0.047
			LPAR4	-0.714	0.047
			SCARNA16	-0.714	0.047
			GAS6-AS1	-0.714	0.047

Gene levels of 15,020 genes were obtained from the transcriptome data of 12 lung fibroblast samples derived from 4 controls and 8 IPF-patients (GSE71351). Correlation with *EPB41L3* mRNA levels were analyzed using Spearman's correlation coefficient analysis.

Table S2. Correlation analysis of *EPB41L3* gene expression intensities with CpG DNA methylation levels of the 12 fibroblasts

Gene Name	Target ID	Region	Loci	Correlation Coefficient	p-value	Transcription factor prediction
SEPB41L3	cg15528411	TSS1500	5545169	0.963	5.E-07	PR B, PR A, GR-alpha
	cg22335490	TSS1500	5544237	0.289	0.362	E2F-1
	cg13034362	TSS1500	5544661	0.214	0.505	-
	cg16924702	TSS1500	5544231	-0.296	0.350	E2F-1
	cg06550462	TSS1500	5544208	-0.249	0.436	TFII-I, GR-alpha
	cg23564700	TSS200	5544169	-0.146	0.651	E2F-1
	cg01673082	TSS200	5544153	0.007	0.983	WT1, E2F-1
	cg16622495	TSS200	5544099	-0.228	0.475	Pax-5, p53, AhR:Arnt
	cg12967001	TSS200	5544089	0.032	0.922	WT1, E2F-1, ETF
	cg23847381	TSS200	5544031	-0.522	0.082	E2F-1
	cg16304950	5'UTR	5543945	-0.289	0.363	E2F-1, AhR:Arnt
	cg02926160	5'UTR	5542743	-0.546	0.066	TFII-I
	cg27082185	5'UTR	5540805	0.853	0.000	Pax-5, p53
	cg14075742	5'UTR	5539428	0.886	0.000	C/EBPbeta
	cg19579167	5'UTR	5543878	0.009	0.978	GR-alpha
	cg07352438	5'UTR	5543548	-0.070	0.828	C/EBPalpha, NF-Y, NF-1, E2F, ENKTF-1, TFII-I, E2F-1
	cg26790372	5'UTR	5542879	0.236	0.460	-
	cg18543270	5'UTR	5543271	0.292	0.358	Pax-5, p53, E2F-1
	cg00027083	5'UTR	5543801	-0.746	0.005	AhR:Arnt, c-Jun
	cg06459104	Body	5456880	0.452	0.140	GR-beta, FOXP3
	cg14090400	3'UTR	5393019	0.907	5.E-05	TFII-I

CpG methylation data were obtained from GSE107226. Correlation with *EPB41L3* mRNA levels were analyzed using Spearman's correlation coefficient analysis.
